# Multiparametric Magnetic Resonance Imaging-based Prostate Specific Antigen Density and PI-RADSv2 score help identify Apical Prostate Cancer

**DOI:** 10.7150/jca.84278

**Published:** 2023-05-15

**Authors:** Cong Huang, Rui Rui, Ninghan Feng, Qun He, Yanqing Gong, Xuesong Li, Shiming He, Liqun Zhou

**Affiliations:** 1 Department of Urology, Peking University First Hospital, Beijing 100034, China.; 2 Institute of Urology, Peking University, National Urological Cancer Center of China, Beijing 100034, China.; 3 Beijing Key Laboratory of Urogenital Diseases (Male) Molecular Diagnosis and Treatment Center, Beijing 100034, China.; 4 Department of Urology, Affiliated Wuxi No. 2 Hospital of Nanjing Medical University, Wuxi 214002, China.; 5 Wuxi Medical College, Jiangnan University, Wuxi 214002, China.

**Keywords:** prostate cancer, magnetic resonance imaging, prostate specific antigen density, PI-RADSv2, apical tumor

## Abstract

**Objective:** To investigate the potential roles of preoperative multiparametric magnetic resonance imaging (mpMRI) in identifying aggressive apical prostate cancer (APCa), thereby helping to facilitate patient counseling and surgical planning.

**Patients and Methods:** We performed a retrospective analysis of 662 patients who underwent radical prostatectomy (RP) between January 2010 to October 2019. All patients underwent a preoperative biopsy and mpMRI of the prostate. APCa was defined as any malignant lesions in the prostatic apex. Clinical, pathological and mpMRI variables were retrieved. Univariate, multivariate, and receiver operating characteristic (ROC) analyses were performed.

**Results:** A total of 214 (32.3%) patients had APCa. Patients presenting APCa were more likely to harbor adverse clinicopathological features (all *p* < 0.05). On univariable analysis, serum prostate-specific antigen (PSA) (*p* < 0.001), mpMRI-based PSA density (PSAD) (*p* < 0.001), Prostate Imaging Reporting and Data System version 2 (PI-RADSv2) score (*p* < 0.001), number of positive cores (*p* < 0.001), percentage of positive cores (*p* < 0.001), max core involvement (*p* < 0.001) and biopsy GG (*p* = 0.001) were significant predictors of APCa. On multivariable analysis, mpMRI-based PSAD ≥ 0.27 ng/ml/cm^3^ (odds ratio [OR]: 2.251, *p* = 0.003), PI-RADSv2 score > 4 (OR: 1.611, *p* = 0.023) and percentage of positive cores (OR: 2.333, *p* = 0.041) were independently predictive of APCa during RP. The AUC values of mpMRI-based PSAD and PI-RADSv2 score were 0.646 (95% Confidence Intervals [CI]: 0.608-0.682) and 0.612 (95% CI: 0.568-0.656), respectively.

**Conclusion:** Preoperative mpMRI-based PSAD and PI-RADSv2 score help identify the presence of APCa and may be useful for surgical decision-making during RP.

## Introduction

Prostate cancer (PCa) is the most common malignant solid tumor among men worldwide^1^. Radical prostatectomy (RP) remains a first-line therapy modality in patients with localized PCa^2^. Positive surgical margin (PSM) is an adverse postoperative characteristic associated with increased risk of biochemical recurrence (BCR)^3^-^6^. The goal of RP is to completely resect the prostate and its primary tumor, while decreasing the complications by preserving the integrity of adjacent structures^7^. The anterior apex of prostate is circumferentially wrapped by neurovascular bundles and membranous urethra, with lacking a true prostatic capsule^8^, ^9^. Thus, apical dissection belongs to one of the most crucial and difficult surgical maneuvers during RP^7^, ^10^. This step has synchronous consequences for oncologic controls via PSM and functional outcomes via urinary continence. Indeed, the prostatic apex is the most frequent location of PSM at RP, ranging from 29% to 39% in previous reports^11^, ^12^. A credible road map showing the presence or absence of APCa preoperatively may help inform urologists when to implement this surgical modification.

Recently, multiparametric magnetic resonance imaging (mpMRI) has emerged as an advantageous tool for identifying PCa lesions and has improved the accuracy of PCa staging^13^. Several researches have suggested that preoperative mpMRI could help predict PSM in men undergoing RP^14^-^16^. In addition, the potential roles of mpMRI information for surgical planning have been formerly emphasized^17^, ^18^. McClure et al. prospectively demonstrated that the utility of mpMRI could help guide the use of nerve-sparing technique during RP although without specific estimation of the apical margin^19^. Consequently, we hypothesized that whether preoperative mpMRI-based information, such as mpMRI-based prostate-specific antigen density (PSAD) and Prostate Imaging Reporting and Data System version 2 (PI-RADSv2) scores^20^, could also be associated with the presence of APCa in patients undergoing RP.

The aim of the present study was to investigate the clinical roles of preoperative mpMRI including mpMRI-based PSAD and PI-RADSv2 scores, in detecting aggressive APCa in men who underwent RP. The mpMRI information may aid urologists to facilitate patient counseling and surgical planning.

## Patients and Methods

### Study population

**Figure 1:**
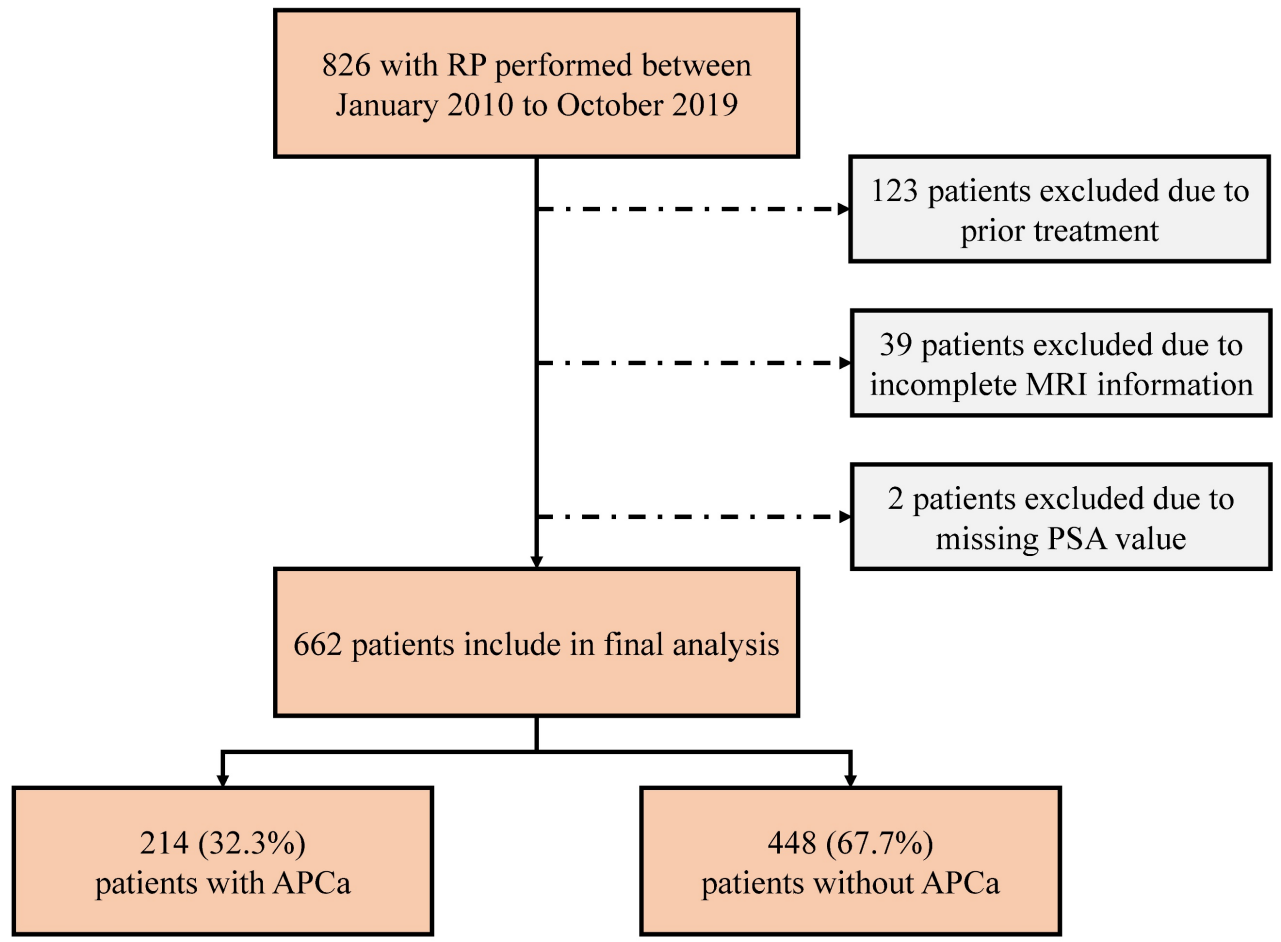
Consort flow diagram for patient selection. RP, radical prostatectomy; MRI, magnetic resonance imaging; PSA, prostate specific antigen; APCa, apical prostate cancer.

The ethics committee of our institute approved this retrospective study, and the require for informed consent was waived. We reviewed the medical charts of 826 patients who underwent laparoscopic or robot-assisted RP from January 2010 to October 2019. All patients underwent systematic transrectal 12- or 13-core prostatic biopsies, with the addition of at least two cognitive fusion targeted biopsies at any area suspected of malignancy by mpMRI or ultrasonography. Preoperative mpMRI were routinely performed for all patients prior to prostate biopsy. Of these patients, 164 were excluded from the analysis due to the following criteria: (1) previously received preoperative radiotherapy and/or androgen deprivation therapy (n = 123), (2) incomplete mpMRI information (n = 39), and (3) incomplete clinicopathological variavles such as PSA value (n = 2). Finally, a total of 662 patients were enrolled in our study (Figure 1).

### MRI protocol

All pelvic mpMRI examinations were performed using 1.5- or 3.0-Tesla scanners with whole-body and pelvic phased array coils (Sigma HDxt, GE Medical System) as previously described^21^. Briefly, the mpMRI acquisition protocol fulfilled the standard of the European Society of Urology Radiology guidelines, and included T1-weighted images (from the whole pelvis) and T2-weighted imaging (in 3 planes, 3 mm slice thickness), diffusion-weighted imaging (3 mm slice thickness with b values of 0, 800, and 1000 s/mm^2^ and dynamic contrast-enhanced imaging (3 mm slice thickness, scan time 3 min, temporal resolution < 9 s). The mpMRI-based prostate volume (PV) was automatically calculated from segmentation of the prostate gland with manual review on a three-dimensional T2-weighted images with high resolution. The mpMRI images were scored and reported according to the PI-RADSv2 criteria by at least two dedicated radiologists (with 10 and 8 years of experience in prostate MRI interpretation, respectively) at our institution.

### Pathological evaluation

All RP surgical specimens were fixed in 10% buffered formaldehyde overnight. Specimens were sliced with standardized multiple transverse 5 mm cuts, using a modified handling technique described previously by the International Society of Urological Pathology (ISUP) consensus conference^22^. Remarkably, the prostate apex of specimens underwent parasagittal separation and was split into two distal apical 5 mm sections. The classifications of patients with or without APCa were defined according to the histologically pathological examinations. The APCa was regarded as any malignant lesions in the apical area, without considering other locations.

### Clinicopathological variables

Both clinical and pathological variables were collected from all patients. The clinical data included age, body mass index (BMI), serum prostate-specific antigen (PSA), digital rectal examination (DRE), PV- determined by mpMRI, clinical T stage (assessed by the 2017 American Joint Committee on Cancer staging system). The mpMRI-based PSAD was calculated by dividing total serum PSA by PV. The pathological characteristics included biopsy and RP specimen Gleason grade group (GG), number of biopsy cores, number of positive cores, percentage of tumor involvement of each biopsy core, pathological T stage, extracapsular extension status, seminal vesicle invasion status, PSM status, and lymph node invasion status. All biopsy and RP specimens were evaluated by two dedicated genitourinary pathologists. The Gleason scoring system followed the ISUP 2005 consensus conference and was adopted to the new Gleason GG system^23^.

### Statistical analysis

Statistical analyses were performed using SPSS version 26.0 (IBM Corporation, Armonk, NY, USA) and MedCalc version 20.21 (MedCalc Software, Mariakerke, Belgium). Quantitative data were presented as median with interquartile range (IQR) or mean ± standard deviations (SD), and the differences between patients with and without APCa were compared using nonparametric Mann-Whitney U tests. Qualitative data were described as frequencies and percentages, and differences were analyzed using chi-square tests. Binary univariate and multivariate logistic regression analyses were conducted to determine the significantly independent variables in the prediction of APCa during RP. Receiver operating characteristic (ROC) curves were produced to evaluate the area under the curve (AUC) and the predictive accuracy. Furthermore, the sensitivities, specificities, positive predictive values (PPVs), and negative predictive values (NPVs) at the optimal cut-off value of mpMRI-based PSAD and PI-RADSv2 scores were analyzed. A *p* value < 0.05 was considered to indicate a statistically significant difference.

## Results

### Baseline characteristics

A total of 662 patients were enrolled in the study. The baseline characteristics are showed in **Table 1**. The median age and serum PSA value were 67 years (IQR: 62-71) and 10.75 ng/ml (IQR: 7.32-16.76). The D'Amico risk classification of low-, medium-, and high-risk groups was 6.1% (40/662), 24.3% (161/662) and 69.6% (461/662), respectively. The median of mpMRI-based PSAD was 0.27 ng/ml/cm^3^ (IQR: 0.16-0.46). Of the entire cohort, the distribution of PI-RADSv2 score was as follows: score 1 to 2 in 101 (15.3%) patients, score 3 in 96 (14.5%) patients, score 4 in 202 (30.5%) patients, and score 5 in 263 (39.7%) patients.

### Clinicopathological comparison between APCa and Non-APCa

**Figure 2:**
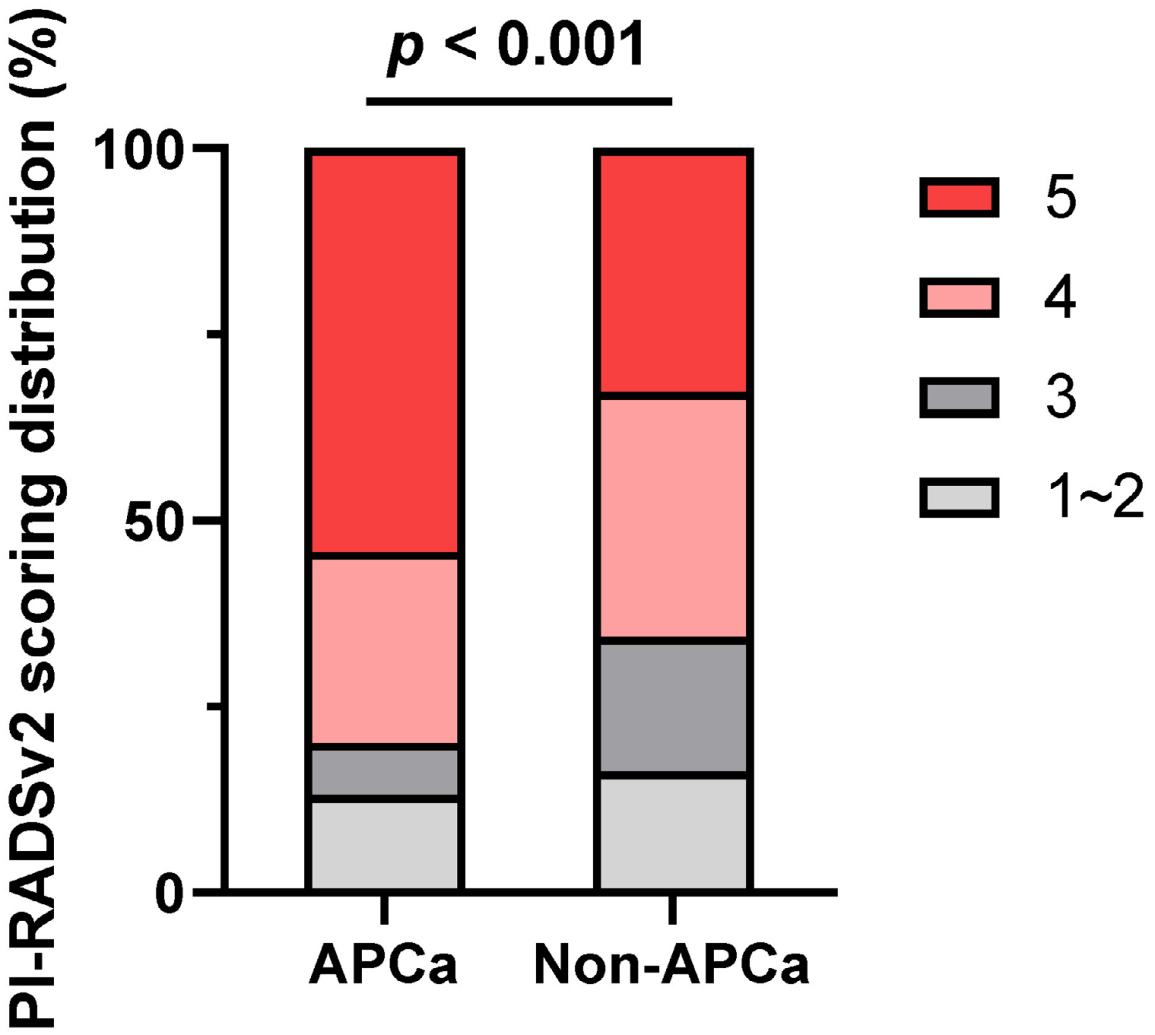
Distribution of PI-RADSv2 scores between patients with APCa and Non-APCa. PI-RADSv2, Prostate Imaging Reporting and Data System version 2; APCa, apical prostate cancer.

Overall, 214 (32.3%) of the cases exhibited APCa and 448 (67.7%) had Non-APCa. Patients with APCa had significantly higher serum PSA (*p* < 0.001), lower BMI (*p* = 0.033), higher mpMRI-based PSAD (*p* < 0.001), and higher PI-RADSv2 score (*p* < 0.001) compared with those who presented Non-APCa (Table 1 and Figure 2). In addition, patients presenting APCa were more likely to harbor unfavorable clinicopathological features such as higher clinical T stage (*p* < 0.001), higher biopsy GG (*p* = 0.011), higher number of positive cores (*p* < 0.001), higher percentage of positive biopsy cores (*p* < 0.001), higher max core involvement (*p* < 0.001), higher pathological T stage (*p* < 0.001), higher pathologic GG (*p* < 0.001), positive surgical margin (*p* < 0.001), extracapsular extension (*p* < 0.001) and seminal vesical invasion (*p* = 0.019). However, there were no significant differences in age (*p* = 0.893), DRE (*p* = 0.115), mpMRI-based PV (*p* = 0.469), number of biopsy cores (*p* = 0.396) and lymph node invasion (*p* = 0.390).

### Uni‑ and multivariable analyses predicting APCa

Binary logistic regression analysis was used to determine independent predictors of APCa at RP. In univariate analysis, the serum PSA (*p* < 0.001), mpMRI-based PSAD (*p* < 0.001), PI-RADSv2 score (*p* < 0.001), number of positive cores (*p* < 0.001), percentage of positive cores (*p* < 0.001), max core involvement (*p* < 0.001) and biopsy GG (*p* = 0.001) emerged as significant predictors of APCa. Age (*p* = 0.531) and DRE (*p* = 0.116) were not significant predictors of APCa at RP. After multivariable analysis, the mpMRI-based PSAD (odds ratio [OR]: 2.251, 95% Confidence Intervals [CI]: 1.330-3.810, *p* = 0.003), PI-RADSv2 score (OR: 1.611, 95% CI: 1.067-2.434, *p* = 0.023), and percentage of positive cores (OR: 2.333, 95% CI: 1.037-5.247, *p* = 0.041) were independently associated with APCa. Max core involvement (*p* = 0.156) and biopsy GG (*p* = 0.656) were not significant independent predictors of APCa in the prostatic apex.

### Receiver operator characteristic curve analysis

**Figure 3:**
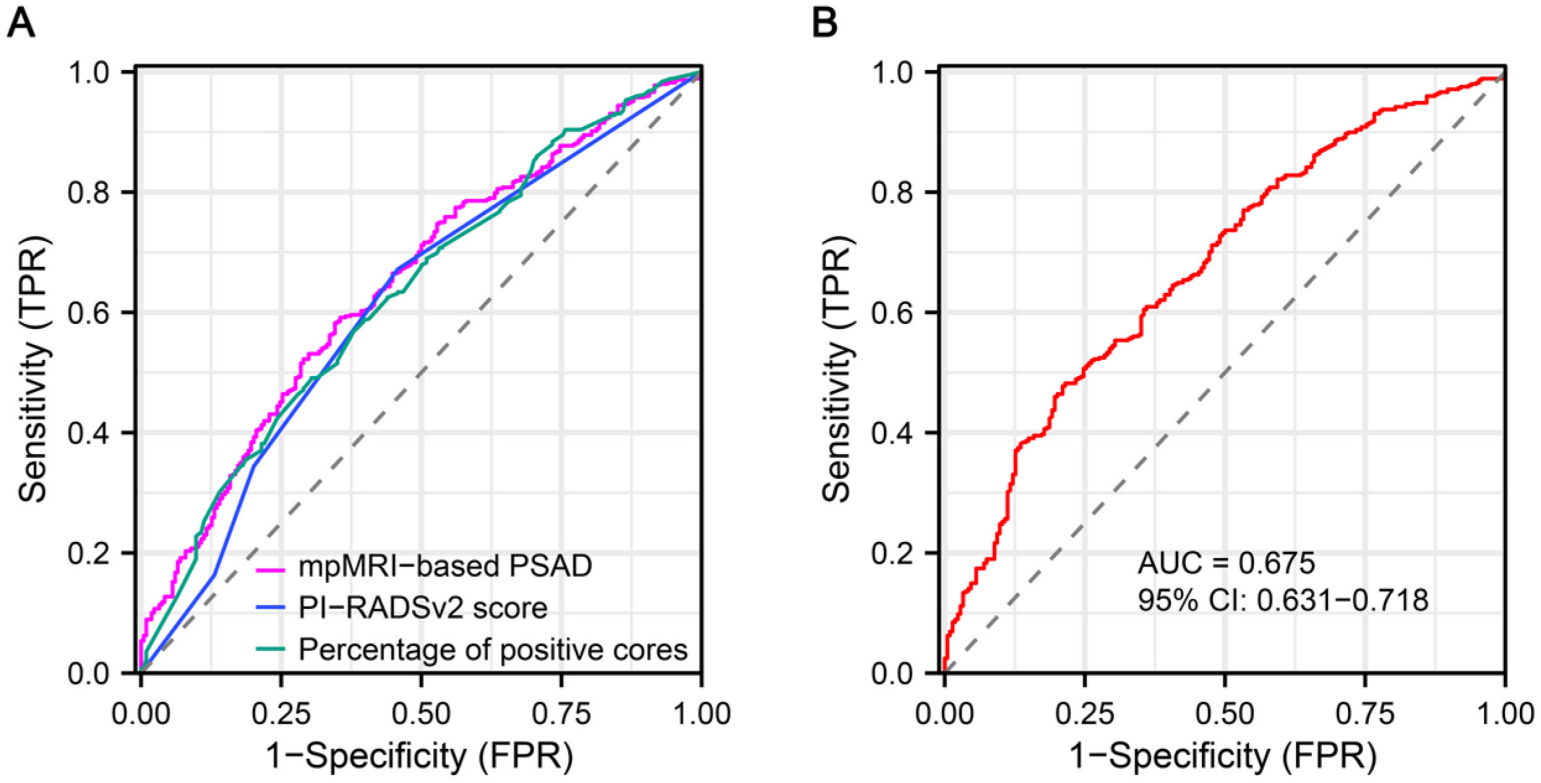
Receiver operator characteristic (ROC) curve shows diagnostic performance of mpMRI-based PSAD and PI-RADSv2 score for predicting APCa at the prostatic apex. APCa, apical prostate cancer; PI-RADSv2, Prostate Imaging Reporting and Data System version 2; mpMRI, multiparametric magnetic resonance imaging.

ROC curves were generated for preoperatively predicting the risk of APCa at RP. The AUC values for mpMRI-based PSAD, PI-RADSv2 score and percentage of positive cores were 64.6, 61.2 and 63.0%, respectively (Figure 3A). Based on ROC analysis, the cut-off for mpMRI-based PSAD to predict APCa was 0.27 ng/m**l/cm^3^**, with a sensitivity of 58.26%, a specificity of 65.42%, a PPV of 77.91%, and an NPV of 42.81%, respectively. The optimum threshold for PI-RADSv2 scores was > 4 for the prediction of APCa. The sensitivity, specificity, PPV, and NPV of PI-RADSv2 scores were, respectively, 67.19%, 54.21%, 75.44% and 44.11%. Moreover, the constructed model including percentage of positive cores, mpMRI-based PSAD and PI-RADSv2 scores exhibited an AUC of 67.5, with a 95% CI of 0.631-0.718 (*p* < 0.001, Figure 3B).

## Discussion

The prostatic apex is one of the most frequent sites for PSM in men undergoing RP^11^, ^24^-^26^. Reasons attributed to this high site-specific PSM rate include the absence of a true anatomic prostatic capsular at the anterior side of the apex, the extreme proximity of the urethral continence mechanism requiring an adjacent surgical margin, and the dilemma in obtaining surgical exposure within a constricted space^27^. Despite the prognostic value of PSM at the apex during RP in predicting residual apical tumors and biochemical failure remains controversial^24^. Several studies have demonstrated significantly higher recurrence rates in patients with apical PSM^3^, ^6^, ^11^. Salomon et al found that patients with positive apical margins were associated with a poorer clinical prognosis in terms of 5-year BCR rates, compared to those with bladder neck or posterolateral positive margins^28^. Nonetheless, Pettus et al showed that apical margin status did not independently predict biochemical recurrence in multivariate analysis^29^. The conflicting results might be explained by the relatively limited sample sizes. In addition, the apical dissection during RP is critical for postoperative urinary continence recovery. It had been suggested that a longer preoperative membranous urethral (MU) length was an independent predictor of continence recovery but simultaneously increased the risk of PSM at the apex^30^, ^31^. Therefore, identifying cancer extension at the apex preoperatively is important to urological surgeons performing RP in order not to compromise the PSM status.

In the current study, APCa was detected in 32.3% (214/662) of all patients after analysis of the RP specimens, comparable to other series. The median serum PSA value was 10.75 ng/ml (IQR: 7.32-16.76). Patients with APCa were found to have higher PSA and more unfavorable pathological features at biopsy. Multivariate analysis revealed that the percentage of positive cores was a significantly independent factor for predicting the presence of APCa. However, many series had shown that systematic biopsy characteristics could not reliably predict an apical PSM^32^, ^33^. Iremashvili et al. demonstrated an inferior NPV of systematic biopsy for detecting the presence of tumor in the apex^34^. Recently, a meta-analysis showed that preoperative MRI exerted significant modification of initial surgical template in one third of PCa patients. The effect occurred increasingly with the rising D'Amico risk category,with 28% in low-, 33% in intermediate-, and 52% in high-risk group^35^. In our study, approximately two-thirds of the patients (69.6%, 461/662) were classified as high-risk in the D'Amico group, which might be due to the lack of a PSA-based PCa screening programme in the Chinese population studied. Thus, the cohort characteristics were rather adverse compared with those in other studies, which is important to consider when interpreting the results.

Nowadays, PSA has been widely utilized as the primary screening and prognostic surveillance index for PCa^36^. However, it still remained controversial because of its limitation on diagnostic specificity. In recent decades, several new parameters related to PSA have been proposed to improve the diagnostic accuracy of PCa, of which PSAD is the most popular^37^. PSAD was recommended as a prognostic biomarker for GG, pathological T stage, active surveillance (AS), and BCR^38^. Busch et al. suggested that PSAD was significantly increased in patients with PSM^39^. Chang et al. found that preoperative PSAD might be a powerful predictor of PSM in patients undergoing RP with PSA levels of less than 10 ng/ml^40^.

Nonetheless, PSAD has an inherent limitation because the calculation of PV with TRUS is inaccurate. It was discovered that the commonly used ellipsoid formula could underestimate PV by more than 10% in most instances^41^. Additionally, interobserver discrepancy and experience also affected how TRUS measurements were made^42^. mpMRI provides far higher soft tissue resolution of the prostate and its surrounding tissues than TRUS. Several studies have demonstrated that mpMRI performed more accurately than TRUS for measuring the actual PV^43^, ^44^. In the present study, mpMRI-based PSAD was independently associated with the presence of APCa during RP. The optimal cut-off value proposed for predicting APCa was 0.27 ng/ml/cm^3^, with an AUC value of 64.6%. The sensitivity, specificity, PPV, and NPV were 58.26%, 65.42%, 77.91%, and 42.80%, respectively. Potential confounders included the disadvantages of the transrectal biopsy scheme and tumor position in the prostate, which were probably related to limited availability in predicting APCa. Miyake et al. showed a significant difference in PSAD between men with and without dorsal apex tumors^45^. Some series found that MRI-based PSAD could also help predict GG upgrade in patients managed with AS^46^, ^47^.

Recently, mpMRI has emerged as a crucial tool for diagnosis, staging, and risk stratification of PCa^48^, ^49^, and its utility for preoperative planning has shown promise^50^. PI-RADSv2 score is designed to standardize image acquisition techniques and interpretation of prostate mpMRI, with worldwide acceptance in academic and community settings^51^, ^52^. Yao et al. demonstrated that preoperative MRI finding was a significant predictor of PSM especially at the prostatic apex^53^. Quentin et al. found that the tumor distance to the MU ≤ 3.5 mm was the strongest MRI predictor for PSM at the apical urethra, with showing the highest accuracy of 95%^16^.

In our study, the PI-RADSv2 score based on mpMRI was a significant predictor of APCa at the prostatic apex, with an AUC value of 61.2%. Moreover, we observed the sensitivity and PPV of PI-RADSv2 for predicting APCa at RP was 67.19% and 75.44%, respectively. Kenigsberg et al. showed that a Likert score > 2 determined by mpMRI helps identify APCa. On multivariate regression analysis, the Likert score and PSA level were significant and independent predictors of tumor in the distal apex^54^. Analogously, Cumarasamy et al. constructed a preoperative multivariable logistic model including PI-RADS ≥ 3 that helps to predict APCa during RP, achieving an AUC value of 72.2%^55^. Furthermore, Veerman et al. suggested that the radiological apical tumor involvement in mpMRI was an independent risk factor for apical PSM and was associated with biochemical recurrence^56^. Our results were at odds with those of previous studies.

The results of the current study suggest that the PI-RADSv2 score is a useful tool for detecting APCa at the apex and could help the surgeon decide when it is appropriate to preserve MU length. A PI-RADSv2 score > 5 should raise suspicion for APCa at the prostatic apex. The limited abilities of mpMRI to diagnose apical lesions have been previously elaborated. One of the disadvantages of mpMRI is that delineation between the mid gland and anterior apex varies according to the reader^57^. Although the PI-RADSv2 scoring system was conducted in an effort to reduce inter-reader variability, it does not provide objective instructions for precise anatomical identification^20^.

There are several limitations to our study that should be considered when interpreting the results. First, it was a relatively small, single-institutional retrospective study with a probable risk of selection bias. Second, most of the patients in the cohort harbored predominantly high-risk diseases, which may limit the catholicity of our findings and possibly undermine the repeatability of the results. Third, interobserver variabilities in interpreting mpMRI findings and pathological review were not performed. In addition, the clinical data collected over quite a long period, during the MRI protocol changed. Finally, we had no information on tumor volume and apical tumor-specific GG. This may help to assess whether the missed apical tumors were clinically significant PCa. Further multicenter prospective work with detailed clinicopathological variables should be performed to confirm and validate the findings in our study.

## Conclusion

Preoperative mpMRI-based PSAD and PI-RADSv2 scores were found to have independently predictive potential for identifying aggressive APCa within the prostatic apex. Our results indicate that mpMRI may help surgeons determine the extent of apical preservation, which is a promising tool for surgical decision-making during RP.

## Figures and Tables

**Table 1:** Baseline characteristics of the study cohort.

Variables	
**Patients, n**	662
**Age, years**	
Median (IQR)	67 (62-71)
Mean ± SD	66.00 ± 6.55
**BMI, kg/m^2^**	
Median (IQR)	24.28 (22.49-26.13)
Mean ± SD	24.41 ± 2.88
**Serum PSA, ng/ml**	
Median (IQR)	10.75 (7.32-16.76)
Mean ± SD	14.65 ± 13.30
**DRE, n (%)**	
Normal	522 (78.9)
Abnormal	140 (21.1)
**D'Amico risk groups, n (%)**	
Low	40 (6.1)
Intermediate	161 (24.3)
High	461 (69.6)
**mpMRI-based PV, ml**	
Median (IQR)	39.00 (30.00-54.53)
Mean ± SD	45.79 ± 24.04
**mpMRI-based PSAD, ng/ml/cm^3^**	
Median (IQR)	0.27 (0.16-0.46)
Mean ± SD	0.38 ± 0.34
**PI-RADSv2 score, n (%)**	
1~2	101 (15.3)
3	96 (14.5)
4	202 (30.5)
5	263 (39.7)
**Number of biopsy cores**	
Median (IQR)	13 (12-14)
Mean ± SD	13.16 ± 2.40
**Number of positive cores**	
Median (IQR)	5 (2-7)
Mean ± SD	5.00 ± 3.32
**Percentage positive biopsy cores, %**	
Median (IQR)	33.33 (16.35-53.85)
Mean ± SD	38.74 ± 25.96
**Max core involvement, %**	
Median (IQR)	70.0 (35.0-85.0)
Mean ± SD	62.07 ± 29.62
**Biopsy GG, n (%)**	
1	165 (24.9)
2	250 (37.8)
3	94 (14.2)
4	76 (11.5)
5	77 (11.6)
**Post-RP GG, n (%)**	
1	59 (8.9)
2	260 (39.3)
3	190 (28.7)
4	58 (8.8)
5	95 (14.4)
**Clinical T stage, n (%)**	
T1	59 (8.9)
T2	540 (81.6)
T3	63 (9.5)
**Pathological T stage, n (%)**	
T2	296 (44.7)
T3	366 (55.3)
**Postoperative pathology, n (%)**	
Positive surgical margin	217 (32.8)
Extracapsular extension	372 (56.2)
Seminal vesicle invasion	152 (23.0)
Lymph nodal involvement	22 (3.3)

IQR, interquartile range; SD, standard deviation; BMI, body mass index; PSA, prostate-specific antigen; DRE, digital rectal examination; mpMRI, multiparametric magnetic resonance imaging; RP, radical prostatectomy; PSAD, prostate-specific antigen density; PI-RADSv2, Prostate Imaging Reporting and Data System version 2; GG, grading group.

**Table 2:** Clinicopathological comparison between patients with and without APCa.

	APCa (n = 214, 32.3%)	Non-APCa (n = 448, 67.7%)	*p* value^#^
**Age, years**			0.893
Median (IQR)	67 (61-71)	67 (62-71)	
Mean ± SD	65.77 ± 6.98	66.11 ± 6.34	
**BMI, kg/m^2^**			0.033^*^
Median (IQR)	24.61 (22.84-26.39)	24.22 (22.19-26.07)	
Mean ± SD	24.75 ± 2.78	24.25 ± 2.91	
**Serum PSA, ng/ml**			< 0.001^*^
Median (IQR)	13.57 (9.27-23.27)	9.63 (6.55-14.91)	
Mean ± SD	19.51 ± 16.56	12.33 ± 9.63	
**DRE, n (%)**			0.115
Normal	161 (75.2)	361 (80.6)	
Abnormal	53 (24.8)	87 (19.4)	
**mpMRI-based PV, ml**			0.469
Median (IQR)	39.30 (30.78-55.05)	39.00 (30.00-53.90)	
Mean ± SD	45.86 ± 21.72	45.76 ± 25.10	
**mpMRI-based PSAD, ng/ml/cm^3^**			< 0.001^*^
Median (IQR)	0.35 (0.21-0.63)	0.23 (0.14-0.39)	
Mean ± SD	0.48 ± 0.38	0.33 ± 0.31	
**PI-RADSv2 score, n (%)**			< 0.001^*^
1~2	28 (13.1)	73 (16.3)	
3	15 (7.0)	81 (18.1)	
4	55 (25.7)	147 (32.8)	
5	116 (54.2)	147 (32.8)	
**Number of biopsy cores**			0.396
Median (IQR)	13 (12-14)	13 (12-14)	
Mean ± SD	12.93 ± 2.29	13.27 ± 2.45	
**Number of positive cores**			< 0.001^*^
Median (IQR)	6 (3-8)	4 (2-7)	
Mean ± SD	5.94 ± 3.46	4.55 ± 3.16	
**Percentage positive biopsy cores, %**			< 0.001^*^
Median (IQR)	42.26 (25.00-69.42)	30.77 (14.29-50.00)	
Mean ± SD	46.88 ± 27.25	34.86 ± 24.41	
**Max core involvement, %**			< 0.001^*^
Median (IQR)	85.0 (50.0-85.0)	70.0 (30.0-85.0)	
Mean ± SD	69.71 ± 26.56	58.42 ± 30.33	
**Biopsy GG, n (%)**			0.011^*^
1	35 (16.4)	130 (29.0)	
2	86 (40.2)	164 (36.6)	
3	35 (16.4)	59 (13.2)	
4	28 (13.1)	48 (10.7)	
5	30 (14.0)	47 (10.5)	
**Post-RP GG, n (%)**			< 0.001^*^
1	8 (3.7)	51 (11.4)	
2	71 (33.2)	189 (42.2)	
3	86 (40.2)	104 (23.2)	
4	11 (5.1)	47 (10.5)	
5	38 (17.8)	57 (12.7)	
**Clinical T stage, n (%)**			< 0.001^*^
T1	31 (14.5)	28 (6.3)	
T2	154 (72.0)	386 (86.2)	
T3	29 (13.6)	34 (7.6)	
**Pathological T stage, n (%)**			< 0.001^*^
T2	64 (29.9)	232 (51.8)	
T3	150 (70.1)	216 (48.2)	
**Postoperative pathology, n (%)**			
Positive surgical margin	137 (64.0)	80 (17.9)	< 0.001^*^
Extracapsular extension	162 (75.7)	210 (46.9)	< 0.001^*^
Seminal vesicle invasion	61 (28.5)	91 (20.3)	0.019^*^
Lymph nodal involvement	10 (4.7)	12 (2.7)	0.390

APCa, apical prostate cancer, IQR, interquartile range; SD, standard deviation; BMI, body mass index; PSA, prostate-specific antigen; DRE, digital rectal examination; mpMRI, multiparametric magnetic resonance imaging; PV, prostate volume; RP, radical prostatectomy; PSAD, prostate-specific antigen density; PI-RADSv2, Prostate Imaging Reporting and Data System version 2; GG, grading group. ^#^Quantitative data were compared using Mann-Whitney U tests. Qualitative data were analyzed using chi-square tests. ^*^statistically significant.

**Table 3:** Uni- and multivariate binary logistic regression analysis predicting APCa.

Variables	Univariate		Multivariate	
	OR (95% CI)	*p* value	OR (95% CI)	*p* value
Age, years	0.992 (0.968-1.017)	0.531	-	-
Serum PSA, ng/ml	1.043 (1.028-1.059)	< 0.001^*^	-	-
mpMRI-based PV, ml	1.000 (0.993-1.007)	0.962	-	-
mpMRI-based PSAD, ng/ml/cm^3^	3.440 (2.111-5.605)	< 0.001^*^	2.251(1.330-3.810)	0.003^*^
DRE	0.732 (0.496-1.080)	0.116	-	
PI-RADSv2 score	2.424 (1.736-3.383)	< 0.001^*^	1.611 (1.067-2.434)	0.023^*^
Number of positive cores	1.133 (1.078-1.190)	< 0.001^*^	-	-
Percentage of positive cores	5.882 (3.115-11.109)	< 0.001^*^	2.333 (1.037-5.247)	0.041^*^
Max core involvement, %	1.014 (1.008-1.020)	< 0.001^*^	1.006 (0.998-1.013)	0.156
Biopsy GG	2.268 (1.381-3.724)	0.001^*^	0.893 (0.544-1.467)	0.656

APCa, apical prostate cancer, OR, odds ratio; CI, Confidence Intervals; PSA, prostate-specific antigen; mpMRI, multiparametric magnetic resonance imaging; PV, prostate volume; PSAD, prostate-specific antigen density; PI-RADSv2, Prostate Imaging Reporting and Data System version 2; GG, grading group. ^*^statistically significant.

**Table 4:** The predictive characteristics of mpMRI-based PSAD and PI-RADSv2 score for predicting APCa.

Variables	AUC (95% CI)	*p* value	Cut-off	Sensitivity (%)	Specificity (%)	PPV (%)	NPV (%)
mpMRI-Based PSAD	0.646 (0.608-0.682)	< 0.001^*^	0.27 ng/ml/cm^3^	58.26	65.42	77.91	42.81
PI-RADSv2 score	0.612 (0.568-0.656)	< 0.001^*^	> 4	67.19	54.21	75.44	44.11
Percentage of positive cores	0.630 (0.585-0.675)	< 0.001^*^	34.96%	56.92	62.15	75.89	40.80
Model	0.675 (0.631-0.718)	< 0.001^*^	-	47.77	78.97	82.63	41.94

APCa, apical prostate cancer, CI, Confidence Intervals; mpMRI, multiparametric magnetic resonance imaging; PSAD, prostate-specific antigen density; PI-RADSv2, Prostate Imaging Reporting and Data System version 2. ^*^statistically significant.
